# Predictability of expansion movements performed by clear aligners in mixed dentition in both arches: a retrospective study on digital casts

**DOI:** 10.1186/s12903-024-04435-y

**Published:** 2024-06-15

**Authors:** Saveria Loberto, Chiara Pavoni, Silvia Fanelli, Letizia Lugli, Paola Cozza, Roberta Lione

**Affiliations:** 1 Phd graduate, Department of Health Science, Unicamillus-Saint Camillus International Medical University, Rome, Italy; 2 Researcher, Department of Health Science, Unicamillus-Saint Camillus International Medical University, Rome, Italy; 3 Research fellow, Department of Health Science, UniCamillus-Saint Camillus International Medical University, Rome, Italy; 4 Professor, Department of Health Science, Unicamillus-Saint Camillus International Medical University, Rome, Italy

**Keywords:** Maxillary expansion, Mandibular expansion, Clear aligner, Predictability, Interceptive treatment, Mixed dentition, Transversal changes, Digital dental casts

## Abstract

**Background:**

to evaluate the predictability of expansion achieved in patients in early mixed dentition treated with Clear Aligners (CA), analyzing the efficiency of the expansion at the end of the first set of aligners and at the end of the therapy in the upper and lower arch.

**Methods:**

36 patients (20 F, 16 M; mean age 8.3 ± 1.5 years) were selected retrospectively from the Department of Orthodontics of the Hospital of Rome “Tor Vergata”. All subjects were treated with CA with no other auxiliaries than attachments. For each patient a standardized sequential expansion protocol was planned for both arches. Digital dental casts were created at three observation periods from an intraoral scanner: prior to treatment (T0), at the end of the first set of aligners (T1), at the end of treatment (T2). The 3D models in planned position determined by the first Clincheck (CC) were obtained for comparison with T1 and T2. Six linear transversal measurements were used to evaluate the dimensional changes and the predictability of expansion movements, comparing T1-CC and T2-CC.

**Results:**

a statistically significant increase within the pre-treatment and the final outcomes for all the variables examined was found. In the upper arch, the greatest level of predictability was detected at the level of the first (46.44%) and second deciduous molar width (44.95%) at T1. The analysis of T2-CC changes showed a significant increase in the percentage of predictability of expansion at the level of the first permanent molars, at mesial (54.86%) and distal (58.92%) width. In the lower arch, a higher percentage of predictability than the upper arch was reported at T1-CC and T2-CC, with the greatest values at the level of second (T1-CC: 48.70%; T2-CC: 75.32%) and first deciduous molar width (T1-CC: 45.71%; T2-CC: 72.75%).

**Conclusions:**

CA can induce significant transversal increments. The predictability of expansion is variable, but it did not exceed the 50% during the first set of aligners. It was necessary to apply refinement set to achieve a good predictability for expansion of about 70%. The expansion in the lower arch was observed to be more predictable than in the upper arch.

## Background

Interceptive orthodontics has the purpose of preventing or improving occlusal problems that might be happening in the transition period from deciduous to permanent dentition [1].

According to the existing literature [2], the malocclusions which should be treated promptly are anterior or posterior crossbites, dento-skeletal Class III, Class II malocclusions with increased overjet, open-bites and dento-basal discrepancies associated with tooth eruption disorders. Most of these malocclusions are characterized by impaired transversal maxillary growth associated with causes of dysfunction, such as functional lateral deviations or oral habits, essential in preventing the onset of structural alterations [3–8].

The transverse maxillary problems result from a symmetric or asymmetric constriction of the basal and/or dentoalveolar arch, with or without posterior crossbites depending upon the severity of the condition [9]. Rapid Maxillary Expansion (RME) is the most effective orthopedic procedure to increase the maxillary transverse dimension in young patients by opening the midpalatal suture [10–12]. However, if the transverse discrepancy has a dentoalveolar etiology, the expansion treatment could be performed exclusively at the dentoalveolar level [13].

The transversal expansion achieved with CA is useful in the correction of non-skeletal malocclusions and to resolve crowding, improving the dental arch form by inducing dento-alveolar changes. In cases of skeletal problems, the treatment must be performed with orthopedic device with skeletal effects, necessarily [17].

Recently, several authors [14–18] evaluated the dimensional changes of the upper arch obtained using CA, reporting an increase of the dentoalveolar maxillary width, with the greatest net improve at the level of upper first deciduous molars (+ 3.7 ± 1.4 mm) and at the level of upper second deciduous molars (+ 3.4 ± 1.6 mm) [16].

To our best knowledge, only two studies in literature analyzed the predictability of expansion in upper and lower arches obtained in patients in mixed dentition.

Gonçalves et al. [19], analyzing 3D digital models of the dental arches of 24 children at pre-treatment, at the digital planning predicted tooth positions, and at achieved tooth positions after treatment with the first round of aligners, reported that the mean efficiency of CA in mixed dentition for the expansion movements in the maxillary arch (mean efficiency: 62.6 ± 18.3%) was slightly greater than in the mandibular arch (mean efficiency: 61.6 ± 32.1%).

More recently, Kim et al. [20] quantified the predictability of arch expansion in children with early mixed dentition treated with CA using digital models obtained at pretreatment, predicted and posttreatment stages for both the maxillary and mandibular arches and assessed the main clinical factors for the predictability of arch expansion. The authors reported that the predictability of arch expansion was significantly higher in the mandibular arch (mean efficiency: 76.25 ± 20.14%) compared to the maxillary arch (mean efficiency: 63.85 ± 21.47%) and significantly lower in the first permanent molars (maxillary 53.4 ± 25.8%, mandibular 69.4 ± 21.5%) than in the other deciduous teeth.

Nevertheless, both studies analyzed the predictability of expansion in a single observation time, specifically at the end of the first set of aligners or at the end of the treatment.

Thus, the aim of this retrospective study was to evaluate the predictability of expansion achieved in patients in early mixed dentition treated with Clear Aligners, analyzing the efficiency of the expansion at the end of the first set of aligners and at the end of the therapy.

The null hypothesis tested was that one set of aligners with a planned expansion about 4–6 mm is necessary to achieve a good predictability of arch expansion in both the maxilla and the mandible.

## Methods

This study project was accepted by the Ethical Committee of the Hospital of Rome “Tor Vergata” (Protocol number: 48/23) and minor subjects’ parents signed a consent form.

The study group comprised a sample of 36 patients (20 females and 16 males), with a mean age of 8.3 years ± 1.5 years, who was treated at the Department of Orthodontics of the Hospital of Rome “Tor Vergata” from January 2022 to December 2023.

The following inclusion criteria were considered for participation in the current study: European ancestry, early mixed dentition stage with fully erupted first permanent molars, posterior transverse discrepancy between maxillary and mandibular arches up to 6 mm, with or without cross-bite, and good compliance with aligners. Exclusion criteria included missing deciduous canines or molars prior to treatment, the presence of multiple or advanced caries, previous orthodontic treatment or use of other auxiliary appliances, tooth agenesis or supernumerary teeth and periodontal diseases.

All patients were treated with Invisalign First System® CA with no other auxiliaries than Invisalign attachments. No enamel interproximal reduction (IPR) [21] or extraction were planned during treatment. Optimized expansion support attachments and optimized retention attachments were automatically placed on the buccal tooth surface by the software. No attachments other than those automatically placed have been used to optimize the expansion protocols designed by the software.

For each patient, a standardized sequential expansion protocol was planned, which included movement of the first permanent molars, followed by simultaneous movement of the deciduous teeth (“molars move first”). The same protocol was programmed for both arches. The amount of arch expansion was of 0.25 mm per stage. Moreover, for upper and lower first permanent molars a simultaneous disto-rotation according to Rickett’s line and 2 degrees of extra buccal root torque were required for each phase of expansion.

The amount of transversal expansion was planned between 4 and 6 mm, defined on a case-by-case basis by taking the cusp relationships as a reference and considering the transverse relationship between the upper and lower first permanent molars. The expansion ClinCheck was planned until the palatal first maxillary molar cusps touched the vestibular first mandibular molar cusps. No overcorrection was digitally designed.

Each patient was recommended to wear the aligners full-time, removing them only during meals and oral hygiene, and to change their aligners every 7 days. In each appointment every 4 weeks, the operator checked aligner fitting, attachment positions and patient’s compliance.

Overall, the mean number of aligners used effectively was 60 for each arch for all treatment.

If new scans were necessary to improve the fitting of the device, the prescription form of the therapy was set up to complete treatment until the same final position of the first approved Clincheck®.

For each patient, three digital dental casts (.stl files) were created at three observation periods from an intraoral scanner iTero® Orthodontic ver. 5.2.1.290 (Align Technology Inc., Santa Clara, CA, USA): prior to treatment (T0), at the end of first set of aligners (T1) and at the end of treatment (T2).

The 3D models in planned position determined by the first Clincheck (CC) were also obtained to perform the comparison with the models at T1 and T2.

Predictability of arch expansion was calculated by comparing planned Clincheck expansion with the achieved expansion obtained at the end of first set of aligners (T1-CC) and planned Clincheck expansion with the achieved expansion obtained at the end of the treatment (T2-CC).

Predictability was defined as the percentage of predicted expansion achieved: predictability (prediction accuracy) = (achieved expansion)/ (planned Clincheck expansion) x 100%.

**Fig. 1:**
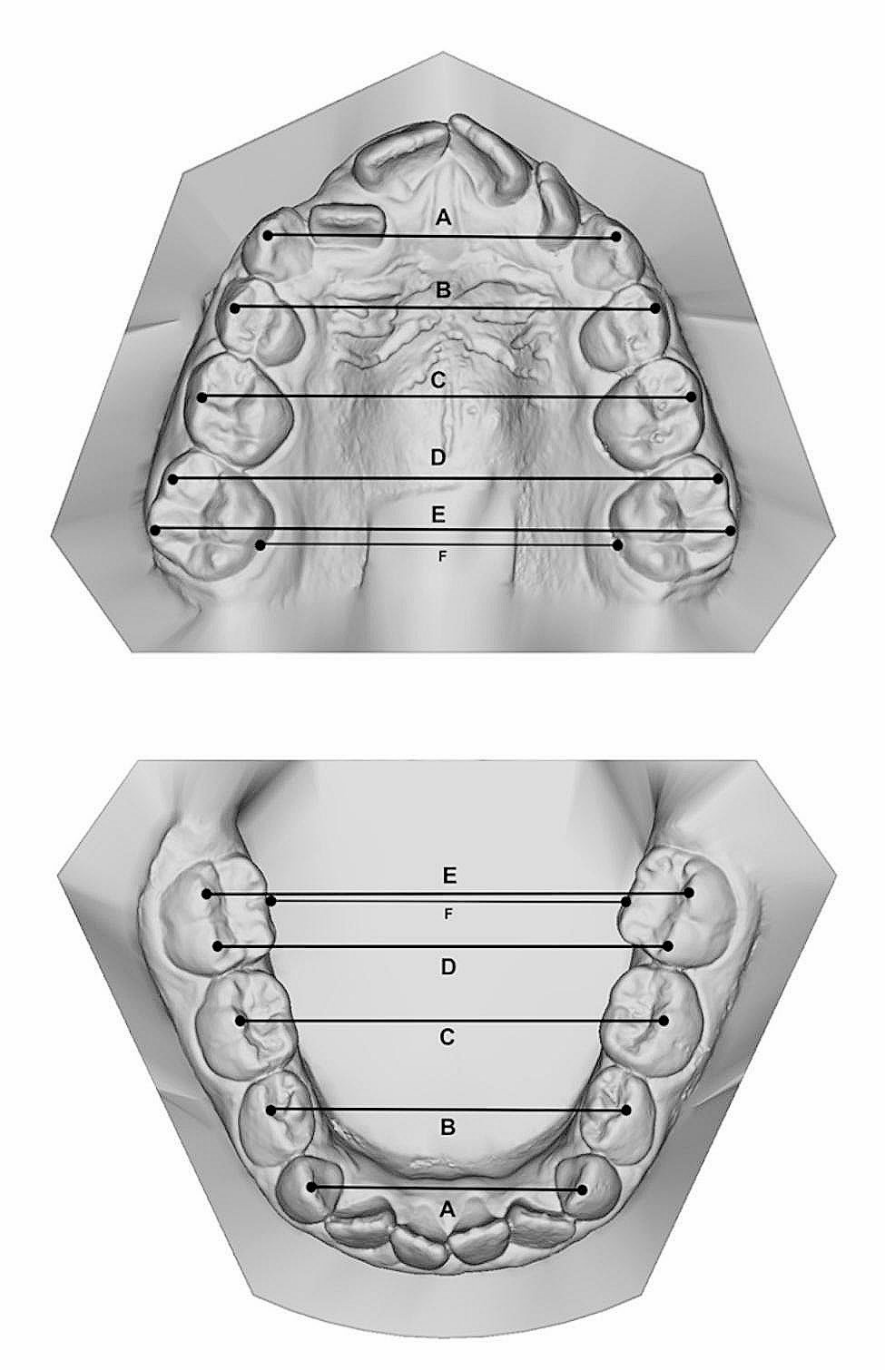
Upper and lower arch widths measured on digital models at the level of deciduous canines’ cusp tips (**A**), vestibular cusp tips of first deciduous molars (**B**), vestibular sulcus of second deciduous molars (**C**), mesiobuccal cusp tips of first permanent molars (**D**), distobuccal cusp tips of first permanent molars (**E**), and sulci of the first permanent molars (**F**)

The following linear transversal measurements were evaluated at all observation times using Viewbox 4 (dHAL software, Kifissia, Greece) for both upper and lower arch:

- Intercanine width (III–III): linear distance between cusp tips of the deciduous canines (A);
- First interdeciduous molar width (IV–IV): linear distance between the vestibular cusp tips of the first deciduous molars (B);
- Second interdeciduous molar width (V–V): linear distance between the sulcus of the second deciduous molars (C);
- First intermolar mesial width (6–6 mesial): linear distance between the mesiobuccal cusp tips of the first permanent molars (D);
- First intermolar distal width (6–6 distal): linear distance between the distobuccal cusp tips of the first permanent molars (E);
- First intermolar transpalatal width (6–6 transpalatal): linear distance between the palatal sulci of the first permanent molars (F) (Fig. 1).

Compliance was evaluated with a 3-point Likert-type scale (poor, moderate, good) [22]. Poor compliance was reported when the patient used the aligners less than 16 h/day; moderate between 16 and 20 h/day; good when the patient used the aligners full time as suggested by the clinicians.

### Statistical analysis

A previous study by Lione et al. [16] was used to calculate the reproducibility and the sample size, which indicated the need for approximately 30 patients to estimate the inter-canine width with a 95% confidence interval (CI), a minimum difference of 1.5 mm and a standard deviation (SD) of 2.0 mm, with a power of 80%. To test inter-examiner reliability, the sample was measured again 2 weeks after the first assessment by another investigator. The reliability of the measurements was assessed by calculating the interclass correlation coefficient (ICC). Sample normality was tested by the Shapiro–Wilk test.

A paired *t*-test was used to compare the two measurements (systematic error, *p* value < 0.05). The magnitude of the random error was calculated by using the method of moment’s estimator (MME) [23].

A Friedman ANOVA for repeated measures followed by the Tukey post-hoc test was selected to compare the T1-T0, T2–T0, T2-T1 changes and to determine the predictability of the Clincheck, comparing T1-CC and T2-CC. The level of significance was set at 5% and Prism 10 (GraphPad Software, LLC) was the chosen software to analyze data.

## Results

No systematic error was found between the repeated measurement values. The ICC test (Intra-class Correlation Coefficient) showed almost perfect agreement with a score of 0.95 for all linear measurements. The analysis of compliance of the treated subjects (use of aligners) showed that none had poor cooperation. As a result, cooperation was good in 50% of treated patients and moderate in the other 50%. The mean time between the T1-T0 was 6.9 ± 1.8 months and between T2-T1 was 8.1 ± 1.4 months, for a time duration of treatment of 15 ± 2.2 months.

Tables 1 and 2 summarize the differences between the linear measurements in the upper and the lower arch dimension detected at the initial (T0), at the end of first set of aligners (T1) and at the end of treatment (T2), the standard deviation (± SD) and the percentage of predictability between planned Clincheck expansion and achieved expansion at the end of first set of aligners (T1-CC) and planned Clincheck expansion and achieved expansion at the end of treatment (T2-CC).

**Table 1:** Statistical comparison of the measurements at T0, T1, T2 and planned ClinCheck expansion (CC) with the Friedman ANOVA for repeated measures followed by the Tukey post-hoc test performed in the upper arch

UPPER	CC-T0		T1-T0		T2-T0		T2-T1		T1-CC		T2-CC	
	Value	SD	Value	SD	Value	SD	Value	SD	Value	Predict. (%)	Value	Predict. (%)
Intercanine width (mm)	4.13	0.45	1.79	0.57	2.78	0.55	0.99	0.55	-2.34 ***	43.3	-1.35 ***	67.3
First interdeciduous molar width (mm)	5.47	0.5	2.54	0.59	3.56	0.58	1.02	0.61	-2.93 ***	46.4	-1.91 ***	65.1
Second interdeciduous molar width (mm)	5.54	0.56	2.49	0.69	3.92	0.62	1.43	0.64	-3.05 ***	44.9	-1.62 ***	70.8
First intermolar mesial width (mm)	5.45	0.7	2.14	0.77	2.99	0.7	0.85	0.71	-3.31 ***	39.3	-2.46 ***	54.9
First intermolar distal width (mm)	3.7	0.56	1.33	0.62	2.18	0.59	0.85	0.58	-2.37 ***	35.9	-1.52 ***	58.9
First intermolar transpalatal width (mm)	3.55	0.58	0.85	0.60	1.76	0.59	0.91	0.59	-2.70 ***	23.9	-1.79 ***	49.6

SD: Standard Deviation; * *p* < 0.05; ** *p* < 0.01; ***: *p* < 0.001;

**Table 2:** Statistical comparison of the measurements at T0, T1, T2 and planned ClinCheck expansion (CC) with the Friedman ANOVA for repeated measures followed by the Tukey post-hoc test performed in the lower arch

LOWER	CC-T0		T1-T0		T2-T0		T2-T1		T1-CC		T2-CC	
	Value	SD	Value	SD	Value	SD	Value	SD	Value	Predict. (%)	Value	Predict. (%)
Intercanine width (mm)	3.3	0.41	1.37	0.43	2.33	0.4	0.96	0.37	-1.93 ***	41.5	-0.97 *	70.6
First interdeciduous molar width (mm)	4.55	0.46	2.08	0.49	3.31	0.46	1.23	0.46	-2.47 ***	45.7	-1.24 *	72.7
Second interdeciduous molar width (mm)	4.62	0.47	2.25	0.48	3.48	0.46	1.23	0.43	-2.37 ***	48.7	-1.14 *	75.3
First intermolar mesial width (mm)	4.07	0.67	1.94	0.72	2,78	0.68	0.84	0.63	-2.13 **	47.7	-1.29 (NS)	68.3
First intermolar distal width (mm)	3.61	0.61	1.56	0.61	2.24	0.61	0.68	0.57	-2.05 **	43.2	-1.37 *	62.0
First intermolar transpalatal width (mm)	3.17	0.41	1.15	0.46	1.6	0.45	0.45	0.45	-2.02 ***	36.3	-1.57 **	50.5

SD: Standard Deviation; * *p* < 0.05; ** *p* < 0.01; ***: *p* < 0.001; NS: not significant.

### Comparison of the measurements in the upper arch

In the period T1-T0, the greatest difference in maxillary arch was found at the level of the first deciduous molars (+ 2.54 ± 0.59 mm), followed by the increase detected at the level of the second deciduous molars (+ 2.49 ± 0.69 mm). When analyzing the movements of first permanent molars, a greater expansion of the intermolar mesial width (+ 2.14 ± 0.77 mm) and distal width (+ 1.33 ± 0.62 mm) than of the intermolar transpalatal width (+ 0.85 ± 0.60 mm) was reported.

**Fig. 2:**
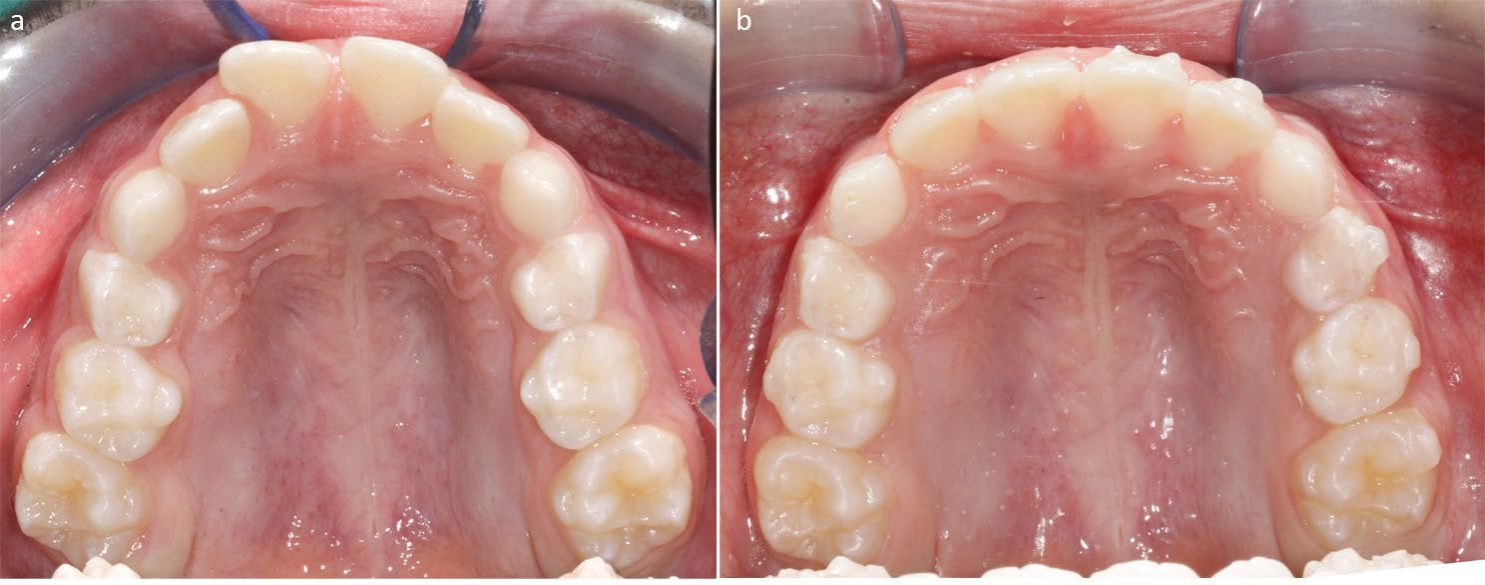
Occlusal views of upper arch at pre-treatment (**a**) and post-treatment (**b**)

In the interval T2-T1, the greatest increase of maxillary width was detected at the level of the second deciduous molars (+ 1.43 ± 0.64 mm), followed by the first deciduous molars (+ 1.02 ± 0.61 mm) and by the deciduous canine (+ 0.99 ± 0.55 mm). (Fig. 2a-b)

From the observation of the changes of the first permanent molars, a greater expansion of the intermolar transpalatal width (+ 0.91 ± 0.59 mm) than of the intermolar mesial width (+ 0.85 ± 0.71 mm) and distal width (+ 0.85 ± 0.58 mm) was highlighted.

As regards the percentage of the effective transversal expansion, in the comparison T1-CC, the greatest level of predictability was detected at the level of the upper first interdeciduous molar width (46.44%; -2.93 mm; *p* ≤ 0.001), followed by the second interdeciduous molar width (44.95%; -3.05 mm; *p* ≤ 0.001) and the interdeciduous canine width (43.34%; -2.34 mm; *p* ≤ 0.001).

As reported in Table 1, about the percentage of the transversal expansion of the first permanent molar, the greatest level of predictability was found at the level of the intermolar mesial width (39.27%; -3.31 mm; *p* ≤ 0.001), followed by the intermolar distal width (35.95%; -2.37 mm; *p* ≤ 0.001) and the intermolar transpalatal width (23.94%; -2.70 mm; *p* ≤ 0.001).

In the comparison T2-CC, the greatest level of predictability was detected on the second interdeciduous molar width (70.76%; -1.62 mm; *p* ≤ 0.001), followed by the interdeciduous canine width (67.31%; -1.35 mm; *p* ≤ 0.001) and the first interdeciduous molar width (65.08%; -1.91 mm; *p* ≤ 0.001). Additionally, about the percentage of the transversal expansion of the first permanent molar, the greatest level of predictability was found at the level of the intermolar distal width (58.92%; -1.52 mm; *p* ≤ 0.001), followed by the intermolar mesial width (54.86%; -2.46 mm; *p* ≤ 0.001) and the intermolar transpalatal width (49.58%; -1.79 mm; *p* ≤ 0.001; Table 1).

### Comparison of the measurements in the lower arch

In the interval T1-T0, the greatest increase of mandibular width was detected at the level of the lower second deciduous molars (+ 2.25 ± 0.48 mm), followed by the first deciduous molars (+ 2.08 ± 0.49 mm) and by the deciduous canine (+ 1.37 ± 0.43 mm). The analysis of the movements of lower first permanent molars showed a greater expansion at the level of the intermolar mesial width (+ 1.94 ± 0.72 mm) and distal width (+ 1.56 ± 0.61 mm) than of the intermolar transpalatal width (+ 1.15 ± 0.46 mm).

**Fig. 3:**
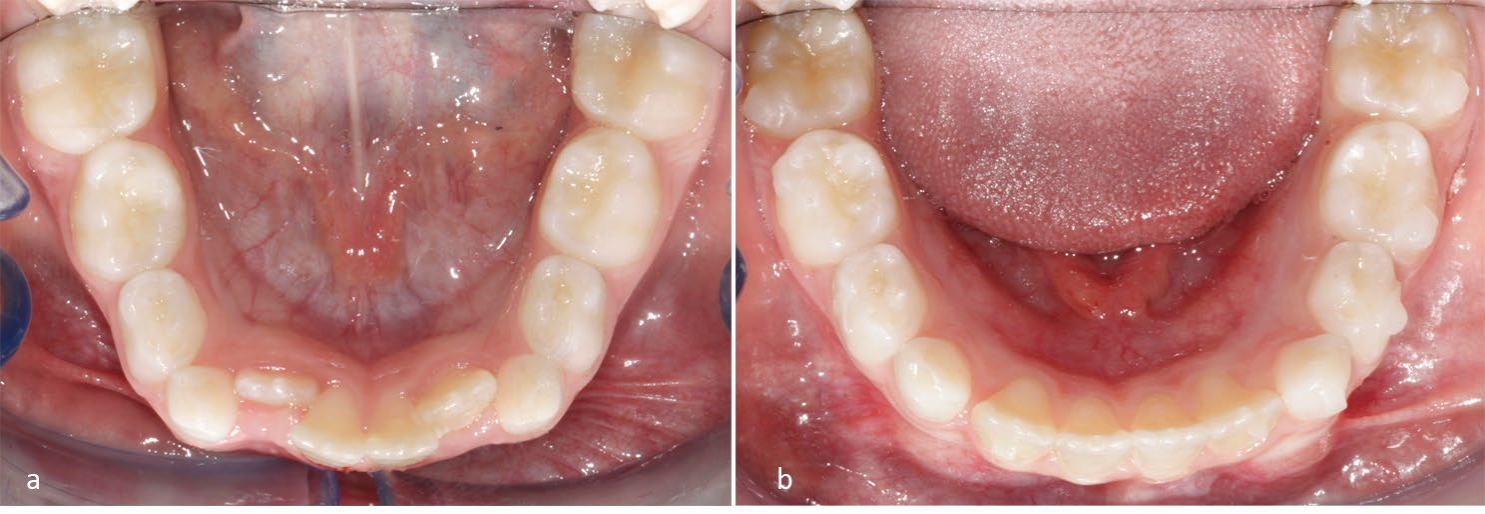
Occlusal views of lower arch at pre-treatment (**a**) and post-treatment (**b**)

In the period T2-T1, the greatest increase of mandibular width was highlighted at the level of the second (+ 1.23 ± 0.43 mm) and first deciduous molars (+ 1.23 ± 0.46 mm) followed by the deciduous canine (+ 0.96 ± 0.37 mm). At the level of first permanent molars, a greater expansion of the intermolar mesial (+ 0.84 ± 0.63 mm) and distal width (+ 0.68 ± 0.57 mm) than of the intermolar transpalatal width (+ 0.45 ± 0.45 mm) was observed. (Fig. 3a-b)

As regards the percentage of the effective expansion in the lower arch, in the comparison T1-CC, the greatest level of predictability for deciduous tooth was detected on the lower second interdeciduous molar width (48.70%; -2.37 mm; *p* ≤ 0.001), followed by the first interdeciduous molar width (45.71%; -2.47 mm; *p* ≤ 0.001). Moreover, about the percentage of the transversal expansion of the first permanent molar, the greatest amount of predictability was detected at the level of the intermolar mesial width (47.67%; -2.13 mm; *p* ≤ 0.01), followed by the intermolar distal width (43.21%; -2.05 mm; *p* ≤ 0.01) and the intermolar transpalatal width (36.28%; -2.02 mm; *p* ≤ 0.001).

In the comparison T2-CC (Table 2), the greatest level of predictability was detected at the level of the second interdeciduous molar width (75.32%; -1.14 mm; *p* ≤ 0.05), followed by the first interdeciduous molar width (72.75%; -1.24 mm; *p* ≤ 0.05) and by the interdeciduous canine width (70.61%; -0.97 mm; *p* ≤ 0.05). Also, about the percentage of the transversal expansion of the first permanent molar, the amount of predictability detected at the level of the intermolar mesial width was 68.30%; -1.29 mm (NS), followed by the intermolar distal width (62.05%; -1.37 mm; *p* ≤ 0.05) and by the intermolar transpalatal width (50.47%; -1.57 mm; *p* ≤ 0.01; Table 2).

## Discussion

Interceptive orthodontic treatment should be predictable, short, and efficient aiming to correct malocclusion traits that would affect function, future dental occlusion and a favorable growth of the jaws [14, 24].

Orthopedic maxillary expansion treatments have been used for more than a century to correct transverse maxillary deficiency by exerting forces at the mid-palatal and intermaxillary sutures in growing patients [17]. On the other hand, dentoalveolar expansion is performed applying the force directly to the teeth and producing a lateral displacement of the upper dento-alveolar structures. Several removable or fixed appliances, including aligners, can be used [14–17].

The purpose of the present retrospective study was to evaluate the effects on the transverse plane of CA in early mixed dentition in a sample of subjects that require dentoalveloar expansion, and to assess the predictability of expansion at the end of the first set of aligners and at the end of treatment for a better understanding of expansion movements during treatment. CA had no effect on the mid-palatal suture and represented an alternative to conventional approaches to obtain maxillary expansion exclusively at the dentoalveloar level. Ideally, all treatment goals should be achieved in the first set of aligners [25], but in clinical practice this condition happens almost never.

Indeed, in our study sample, each patient required at least two sets of additional aligners, in agreement with Pinho et al. [14], who reported that only 69% of malocclusions traits can be solved within the first set of aligners.

In particular, dentoalveolar expansion correction of non-skeletal constricted arches with initial negative molars torque was reported to reach 80% of the planned movements with the initial series of aligners [14]. According to literature, several Authors [14, 16, 20] reported that additional aligners are needed to overcome challenges such as the breakage of aligners or the need to improve the fit of aligners due to tooth loss and eruption. Although Invisalign First® clear aligners are specifically designed to treat in mixed dentition, the management of short clinical crowns and of tooth replacement was complex, and consequently the efficiency of the device may also be affected. Therefore, the use of additional aligners can represent a treatment strategy suitable to achieve all the objectives of therapy and to manage the development of dentition.

Invisalign® First is designed to achieve up to 8 mm expansion. However, as described by Pinho et al. [14], a maxillary first permanent molar transversal distances evaluation suggested that dentoalveolar expansion between 3 and 4 mm was a predictable movement, instead an expansion movement of > 4 to 6 mm with negative molar torque is considered to be an intermediate correction. An expansion greater than 6 mm was considered a treatment having a skeletal component and therefore to be treated with orthopaedic devices. Furthermore, expansion values between 4 and 6 mm are the most requested by clinicians in interceptive treatment performed by CA [14] and applicable for this sample.

To our best knowledge, only few articles evaluated the expansion predictability in growing patients presenting with mixed dentition and constricted maxillary arch [14, 16, 19, 20].

Gonçalves et al. [19] compared the results of the expansion obtained after the first set of aligners in both arches, pointing out an efficiency of maxillary transversal expansion of 55,2% on deciduous canines, 60.7% on first deciduous molars and 63.3% on second deciduous molars at the end of the first round of aligners. The predictability of expansion at the level of first upper permanent molars was 61,1%. More recently, Kim et al. [20] evaluated the predictability at the end of the expansion treatment on a sample of 90 patients in mixed dentition. The authors found a mean predictability of maxillary tooth expansion of 71.1% for deciduous canines, 67.5% for first deciduous molars, 65.2% for second deciduous molars and 53.4% for first permanent molars at the end of the treatment.

In the present study, the amount of movement achieved compared to the amount of movement programmed resulted in less than 47% for all measurements at the end of the first set of aligners, with a greater improve at the end of interceptive treatment, in the upper and lower arch.

At T2 observation period, the highest predictability was observed at the level of second deciduous molars (70.76%) followed by intercanine width (67.31%). The predictability of expansion movements was detected to be of about 56% at the level of first permanent molars (54.86% on first intermolar mesial width and 58.92% on first intermolar distal width).

The lower predictability at the level of the first upper permanent molars can be explained by several factors, including differences in root surface area, aligner material limitations in exerting appropriate force magnitudes for different teeth, differences in cortical bone thickness, occlusal load and soft tissue pressures [26, 27]. Furthermore, the terminal part of the aligners is the most flexible part and, therefore, a lower expansion force can be exerted [18].

Regarding the lower arch, our results are in agreement with Kim et al. [20], who described the maxillary arch expansion less predictable than mandibular expansion in mixed dentition.

Generally, the amount of planned transversal expansion in the upper arch is greater than in the lower. When the maxilla is narrow, the lower curve of Wilson is accentuated as result of dental compensations [28, 29].

Therefore, the sequential expansion protocol involves only a vestibular tipping movement of the crowns in the mandibular arch.

In our study, the predictability of expansion at the end of first set of aligners was 45.71% on first deciduous molars, 48.70% on second deciduous molars and 41.52% on deciduous canines, and of about 45.44% (46.67% on first intermolar mesial width and 43.21% on first intermolar distal width) at the level of lower permanent molars.

These results are in agreement with Gonçalves et al. [19], who found a predictability of expansion on deciduous canines of 52.2%, 46.2% on first deciduous molars, 59.9% on second deciduous molars and 66.8% on first permanent molars.

As for the maxillary arch, the predictability highly increased at the end of treatment, with the greatest percentage at the level of the second interdeciduous molar width (75.32%), followed by the first interdeciduous molar width (72.75%) and by the interdeciduous canine width (70.61%). About the percentage of the transversal expansion of the lower first permanent molars, the greatest amount of predictability was detected at the level of the intermolar mesial width of about 68.30% (intermolar distal width 62.05%; intermolar transpalatal width 50.47%).

Kim et al. [20] detected higher percentages of predictability of expansion on deciduous teeth at the end of treatment than the results of this study, reporting 81.1% for deciduous canines, 81.2% for first deciduous molars, 77.8% for second deciduous molars. The predictability on first permanent molars (69.4%) was similar to the value found in this research.

Despite the discrepancy between the percentages, all studies collected amount of expansion significantly lower in the first permanent molars than in the other deciduous teeth [19, 20].

One limitation of the present investigation was the retrospective nature of the paper and the absence of a control group. Despite these limitations, the present study allowed to explain the need of additional set of aligners to achieve a good predictability.

## Conclusions

Clear aligners treatment in early mixed dentition can determine dento-alveolar arch expansion (predictability: 39% at T1 and 61% at T2 in upper arch; 44% at T1 and 67% at T2 in lower arch). However, the null hypothesis was rejected: refinement set of aligners are needed to achieve a good predictability of expansion of about 70% in both arches. The expansion movements in the lower arch were observed to be more predictable than the expansion movements in the upper arch.

## Data Availability

The datasets used and/or analyzed during the current study are available from the corresponding author on reasonable request.

## Abbreviations

CA: Clear Aligners
RME: Rapid Maxillary Expansion
IPR: enamel interproximal reduction
